# Mesoporous Silica Nanoparticle-Coated Microneedle Arrays for Intradermal Antigen Delivery

**DOI:** 10.1007/s11095-017-2177-4

**Published:** 2017-05-23

**Authors:** Jing Tu, Guangsheng Du, M. Reza Nejadnik, Juha Mönkäre, Koen van der Maaden, Paul H. H. Bomans, Nico A. J. M. Sommerdijk, Bram Slütter, Wim Jiskoot, Joke A. Bouwstra, Alexander Kros

**Affiliations:** 1 Department of Supramolecular & Biomaterials Chemistry, Leiden Institute of Chemistry (LIC), Leiden University, Leiden, 2300 RA The Netherlands; 2 Division of Drug Delivery Technology, Cluster BioTherapeutics, Leiden Academic Centre for Drug Research (LACDR), Leiden University, Leiden, 2300 RA The Netherlands; 3 Laboratory of Materials and Interface Chemistry & Center of Multiscale Electron Microscopy, Department of Chemical Engineering and Chemistry, and Institute for Complex Molecular Systems, Eindhoven University of Technology, Eindhoven, 5600 MB The Netherlands; 4 Division of Biopharmaceutics, Cluster BioTherapeutics, Leiden Academic Centre for Drug Research (LACDR), Leiden University, Leiden, 2300 RA The Netherlands

**Keywords:** intradermal antigen delivery, lipid bilayer, mesoporous silica nanoparticles, pH-sensitive microneedle arrays

## Abstract

**Purpose:**

To develop a new intradermal antigen delivery system by coating microneedle arrays with lipid bilayer-coated, antigen-loaded mesoporous silica nanoparticles (LB-MSN-OVA).

**Methods:**

Synthesis of MSNs with 10-nm pores was performed and the nanoparticles were loaded with the model antigen ovalbumin (OVA), and coated with a lipid bilayer (LB-MSN-OVA). The uptake of LB-MSN-OVA by bone marrow-derived dendritic cells (BDMCs) was studied by flow cytometry. The designed LB-MSN-OVA were coated onto pH-sensitive pyridine-modified microneedle arrays and the delivery of LB-MSN-OVA into *ex vivo* human skin was studied.

**Results:**

The synthesized MSNs demonstrated efficient loading of OVA with a maximum loading capacity of about 34% and the lipid bilayer enhanced the colloidal stability of the MSNs. Uptake of OVA loaded in LB-MSN-OVA by BMDCs was higher than that of free OVA, suggesting effective targeting of LB-MSN-OVA to antigen-presenting cells. Microneedles were readily coated with LB-MSN-OVA at pH 5.8, yielding 1.5 μg of encapsulated OVA per microneedle array. Finally, as a result of the pyridine modification, LB-MSN-OVA were effectively released from the microneedles upon piercing the skin.

**Conclusion:**

Microneedle arrays coated with LB-MSN-OVA were successfully developed and shown to be suitable for intradermal delivery of the encapsulated protein antigen.

**Electronic supplementary material:**

The online version of this article (doi:10.1007/s11095-017-2177-4) contains supplementary material, which is available to authorized users.

## Introduction

Vaccination is regarded as one of the most promising strategies for reducing mortality and improving human health (1,2). Most of the current vaccines are delivered by intramuscular or subcutaneous injection, which have inherent limitations, such as the risk of infections induced by reusing needles and syringes and the needle fear of children and patients. Therefore, new needle-free, easy to use and effective vaccination methods are urgently needed. One of these potential methods is microneedle-mediated intradermal vaccination (3).

Intradermal vaccination is attractive because the skin is easily accessible and harbors a large number of immune cells, such as dendritic cells (DCs) (1,4). Microneedles are micron-sized structures with a length of less than 1 mm which can be used to overcome the skin barrier located in the top layer of the skin. As these needles do not penetrate to the depths where nerve endings reside, coating of antigens on microneedles enables minimally-invasive and pain-free delivery of vaccines into skin (5–7). A major challenge however, is the limited dose that can be delivered with coated microneedles. In an effort to improve coating efficiency, our lab designed pH-sensitive pyridine-modified microneedles with a surface p*K*_a_ below physiological pH, which allows the adsorption of negatively-charged proteins at slightly acidic conditions (pH 5.8) and their release at neutral pH (pH 7.4). In our previous study, intradermal immunization using pH-sensitive microneedles coated with 5.7 μg OVA was compared to conventional subcutaneous or intradermal immunization (8,9). Microneedle-mediated immunization led to comparable T-cell responses but 10-fold lower IgG responses when compared to conventional subcutaneous or intradermal immunization. Possible strategies to further improve the immunogenicity of vaccines by the intradermal route could be adding an adjuvant or using nanoparticles to deliver the antigens (2,6,10–13).

The adjuvanticity of nanoparticles is attributed to their capability of protecting antigens from degradation, forming a depot at the site of injection, and facilitating antigen uptake by DCs (14). A variety of nanosized vaccine delivery systems have been developed, such as polymeric nanoparticles (15), emulsions (16), and lipid-based nanoparticles (15,17). Recently mesoporous silica nanoparticles (MSNs) have gained significant attention as drug delivery vehicles because of their controlled size and mesostructure, excellent *in vivo* biocompatibility, and their large surface area and pore volume, enabling the efficient loading of active small molecules or proteins (2,18–21).

**Scheme 1:**
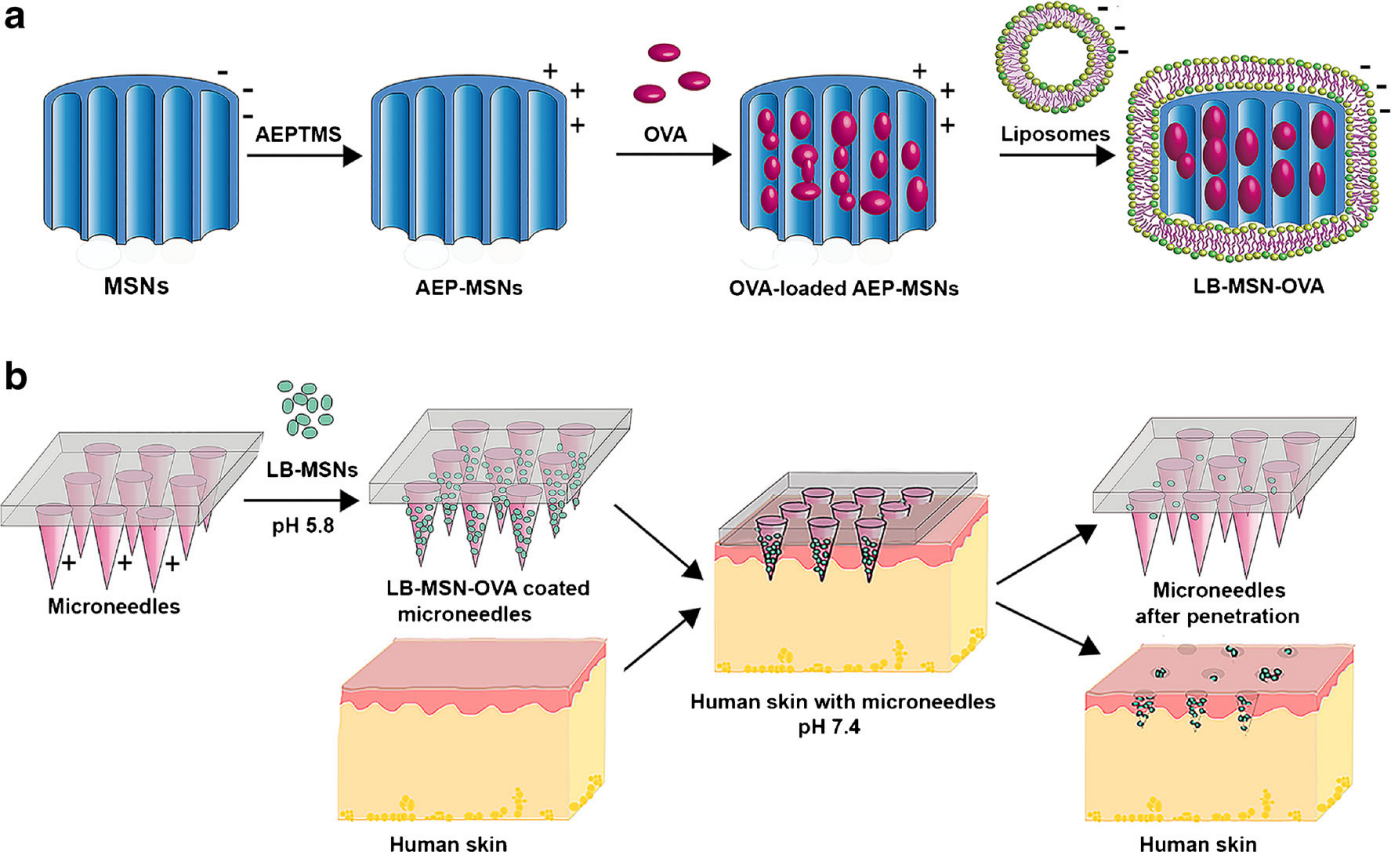
Preparation and application of pH-sensitive microneedle arrays coated with LB-MSN-OVA. (**a**) Encapsulation of OVA into AEP-MSNs, followed by fusion of liposomes (composed of DOPC/DOPS/cholesterol), resulting in LB-MSN-OVA. (**b**) Adsorption of LB-MSN-OVA onto pH-sensitive microneedles and penetration of microneedles into human skin, resulting in a pH shift and delivery of LB-MSN-OVA into the viable epidermis and dermis.

Herein, we report a new intradermal delivery system, which synergistically integrates the advantages of nanoparticles and microneedles by coating pH-sensitive microneedles with antigen-loaded, lipid bilayer-covered MSNs. As a model antigen, OVA was used. This protein is negatively charged (pI of 4.9) (22) at pH 7.4. For the delivery of OVA, a novel type of ultrafine MSNs with large pores (~10 nm in diameter) was synthesized with a positive surface charge (AEP-MSNs), resulting in efficient loading of OVA in the AEP-MSN pores. To enhance the colloidal stability of OVA-loaded AEP-MSNs and generate a negative surface charge, a negatively charged lipid bilayer (LB) was assembled at the AEP-MSN surface and the lipid-coated and OVA-loaded AEP-MSNs are referred to as LB-MSN-OVA (23–25). This method synergistically combines features of liposomes and MSNs and has been reported to address the multiple challenges including stability, targeting and multicomponent delivery (24,25). The designed LB-MSN-OVA were coated onto pH-sensitive pyridine-modified silicon microneedles by electrostatic interactions between the pyridine groups and the LB-MSN-OVA at low ionic strength. Piercing the LB-MSN-OVA coated microneedles into *ex vivo *human skin resulted in the successful release of the nanoparticles due to a shift in pH from 5.8 to 7.4 (Scheme 1).

## Materials and Methods

### Materials

Tetraethyl orthosilicate (TEOS, 98%), sulfuric acid (96%–98%), hydrochloric acid (36%–38%), (3-aminopropyl)triethoxysilane (APTES, 99%), 4-pyridinecarboxaldehyde (97%), sodium cyanoborohydride (95%), 3-[2-(2-aminoethylamino)ethylamino] propyltrimethoxysilane (AEPTMS, technical grade), Ovalbumin (OVA, ≥98%), 1,3,5-trimethylbenzene (TMB, 97%), Pluronic P123 (EO_20_PO_70_EO_20_, Mn ∼ 5800 g/mol), and cholesterol (≥99%) were purchased from Sigma-Aldrich (Zwijndrecht, the Netherlands). Fluorocarbon surfactant FC-4 was purchased from Yick-Vic Chemicals & Pharmaceuticals (HK) Ltd. 1,2-dioleoyl-sn-glycero-3-phosphocholine (DOPC), 1,2-dioleoyl-sn-glycero-3-[phospho-_L_-serine](sodium salt) (DOPS), and 1,2-dioleoyl-sn-glycero-3-phosphoethanolamine-N-(lissamine rhodamine B sulfonyl) (ammonium salt) (DOPE-LR) were purchased from Avanti Polar Lipids Inc. (Alabaster, AL). Hydrogen peroxide (30%) and ethylenediaminetetraacetic acid (EDTA) were purchased from Fluka (Steinheim, Germany). Toluene (≥99.7%) was purchased from Biosolve (Valkenswaard, the Netherlands). Alexa Fluor®488 ovalbumin conjugates (OVA-AF488), anti-CD40-FITC, anti-CD80-PE and anti-CD86-APC were purchased from Thermo Fisher Scientific (Waltham, MA). Sterile phosphate buffered saline (PBS, 163.9 mM Na^+^, 140.3 mM Cl^−^, 8.7 mM HPO_4_^2−^, 1.8 mM H_2_PO_4_^−^, pH 7.4) was obtained from Braun (Oss, the Netherlands). All the other chemicals used are of analytical grade and used without further purification. Milli-Q water (18.2 MΩ/cm, Millipore Co., USA) was used for the preparation of solutions. 1 mM phosphate buffer (PB) with a pH of 7.4 was prepared in the lab. Silicon microneedle arrays with 576 microneedles per array on a back plate of 5 × 5 mm^2^ and a length of 200 μm per microneedle were kindly provided by Robert Bosch GmbH (Stuttgart, Germany).

### Synthesis of MSNs and Amino-Functionalized MSNs (AEP-MSNs)

Mesoporous silica nanoparticles were synthesized according to a published procedure with modifications (26). Briefly, surfactant Pluronic P123 (0.5 g) and FC-4 (1.4 g) were dissolved in HCl (80 mL, 0.02 M), followed by the introduction of TMB (0.48 mL). After stirring for 6 h, TEOS (2.14 mL) was added dropwise. The resulting mixture was stirred at 30°C for 24 h and transferred to an autoclave at 120°C for 2 days. Finally, the solid product was isolated by centrifugation, and washed with ethanol and Milli-Q water. The organic template was completely removed by calcination at 550°C for 5 h.

To prepare cationic MSNs, AEPTMS in absolute ethanol (4 mL, 20 wt%) was incubated with MSNs (100 mg) overnight at room temperature. The desired AEP-MSNs were collected by centrifugation and washed with ethanol to remove unreacted AEPTMS.

### Characterization of MSNs and AEP-MSNs

Morphology of MSNs and AEP-MSNs was visualized by transmission electron microscopy (TEM) using a JEOL 1010 instrument (JEOL Ltd., Peabody, MA) with an accelerating voltage of 70 kV. To prepare the samples, several droplets of nanoparticle suspension (1 mg/ml) were put on a copper grid, dried overnight and coated with carbon.

Nitrogen adsorption-desorption isotherms of samples were obtained with a TriStar II 3020 surface area analyzer (Micromeritics, Norcross, GA). Before each measurement, MSNs were outgassed in the vacuum (below 0.15 mbar) at 300°C for 16 h, while AEP-MSNs were outgassed at room temperature. The specific surface areas were calculated from the adsorption data in the low pressure range using the Brunauer-Emmett-Teller (BET) model (27). The pore size distribution was determined following the Barrett-Joyner-Halenda (BJH) model. Thermogravimetric analysis (TGA) with a Perkin Elmer TGA7 (Waltham, MA) was used to measure the amount of amine-containing groups on the surface of AEP-MSNs. All the samples were tested under an air atmosphere from 25°C to 800°C at a heating rate of 10°C/min.

### Encapsulation of OVA in AEP-MSNs

For loading of OVA into AEP-MSNs, OVA (0.5 mL, 0.5 mg/mL‚ 1 mM PB) and AEP-MSN (0.5 mL, 2 mg/mL, 1 mM PB) were mixed and incubated in Eppendorf mixer (400 rpm, 25°C, Nijmegen, the Netherlands) for different time periods (0, 0.5, 1, 2, 4, 8 and 24 h). After incubation, the suspensions were centrifuged and the encapsulation efficiency (EE%) of OVA was determined by measuring the difference in its intrinsic fluorescence intensity with a plate reader (Tecan M1000, Männedorf, Switzerland) (excitation wavelength = 280 nm and emission wavelength = 320 nm) in the supernatant before and after the encapsulation.

To determine the maximum loading capacity (LC%) of OVA in AEP-MSNs, the AEP-MSNs (2 mg/mL) were mixed with different initial concentrations of OVA (ranging from 0.25, 0.5, 1, 1.5, 2 to 3 mg/mL) and incubated in an Eppendorf mixer (400 rpm, 25°C) for 0.5 h. Next, the suspensions were centrifuged at 9000 g for 5 min. The EE% of OVA was determined by measuring the difference in their intrinsic fluorescence intensity in the supernatant before and after the encapsulation with a plate reader (Tecan M1000).

The EE% and LC% were calculated as below:

1 $$ \mathrm{EE}\%=\frac{t_{ova}-{f}_{ova}}{t_{ova}}\times 100\% $$

2 $$ \mathrm{LC}\%=\frac{t_{ova}-{f}_{ova}}{OVA\  loaded\  AEP- MSNs}\times 100\% $$

Where *t*_ova_ represents the total content of OVA, and *f*_ova_ is the content of free OVA (OVA in the supernatant).

### Preparation of Liposomes

Liposomes were prepared by dispensing stock solutions of DOPC, DOPS and cholesterol in a molar ratio of 7/1/2 into scintillation vials. All lipids were dissolved in chloroform. A lipid film was generated by slow evaporation of chloroform in the vial under a nitrogen flow and dried under vacuum overnight. The lipid film was rehydrated by the addition of PB (1 mL, 1 mM, pH 7.4) and the mixture was vortexed for 10 s to form a cloudy lipid suspension. The obtained suspension was sonicated in a water bath for 10 min. The resulting clear liposomes dispersions were stored at 4°C. To obtain fluorescent liposomes, a fluorescently labeled lipid (DOPE-LR) was incorporated into the liposomes by adding the lipids at 1 wt% DOPE-LR to the lipid solution prior to liposome formation.

### Preparation of LB-MSN-OVA

To prepare LB-MSN-OVA, OVA (0.5 mL, 0.25 mg/mL) solution in PB (1 mM, pH 7.4) was first transferred into a 2-mL Eppendorf tube, followed by the addition of AEP-MSNs (0.5 mL, 1 mg/mL) in PB (1 mM, pH 7.4) and liposomes (0.5 mL, 2 mg/mL) in PB (1 mM, pH 7.4). The resulting mixture was incubated in the Eppendorf mixer for 1.5 h (400 rpm, 25°C). The particles were collected and excess liposomes and OVA were removed by centrifugation (9000 g, 5 min). The encapsulation efficiency of OVA was determined by measuring the difference in their intrinsic fluorescence intensity in the supernatant before and after the encapsulation on a Tecan M1000 plate reader. All experiments were performed in triplicate. For the uptake study of LB-MSN-OVA in dendritic cells, OVA-AF488 was used to prepare LB-MSN-OVA.

### Characterization of LB-MSN-OVA

The hydrodynamic size distribution was measured with dynamic light scattering (DLS) using a Malvern Nano-zs instrument (Worcestershire, UK). Samples were diluted with 1 mM PB (pH 7.4) and measured 3 times each with 10 runs at 25°C. The zeta potential was measured by laser Doppler velocimetry using the same instrument. Samples were diluted with 1 mM PB (pH 7.4) and measured 3 times with 20 runs.

The size distribution was also measured by NanoSight LM20 (NanoSight Ltd., Amesbury, UK). Samples were injected into chamber by an automatic pump (Harvard Apparatus, catalog no. 98–4362, Holliston MA). The samples were diluted to 5 μg/ml with 1 mM PB (pH 7.4) and measured at 25°C. A 90-s video was captured with the shutter set at 1495 and the gain at 680. The data was analyzed by NTA 2.0 Build 127 software.

Imaging of LB-MSN-OVA was performed by using a CryoTitan (FEI Corp, Hillsboro, OR) operating at 300 kV and equipped with a field emission gun (FEG). Cryo-samples were prepared from a 3 μL droplet of sample solution placed on the grid inside the Vitrobot™ chamber at 100% relative humidity and 20°C. Prior to use the TEM grids were glow discharged by a Cressington 208 carbon coater to render them hydrophilic. The samples were blotted to remove excess solution and vitrified by using an automated vitrification robot (Vitrobot™ Mark III, FEI Corp).

### OVA Release Studies from AEP-MSNs and LB-MSN-OVA

To study the influence of ionic strength on the release of OVA from AEP-MSNs, phosphate buffer (PB, 1 mM Na_2_HPO_4_ and 1 mM NaH_2_PO_4_ were mixed at molar ratio of 5:2, pH 7.4) with various concentrations of NaCl (0, 0.9, 1.8, 3.6, 7.2, 14.4 and 28.8%, m/v) were prepared. AEP-MSNs loaded with OVA (1 mg, based on the mass of AEP-MSNs) were dispersed in one of the buffers (1 mL) mentioned above. The suspensions were kept in the Eppendorf mixer for 0.5 h (400 rpm, 37°C), followed by centrifugation (9000 g, 5 min) to collect the supernatant. The amount of released OVA in the buffer was quantified by measuring the intrinsic fluorescence intensity of OVA with a Tecan M1000 plate reader. The released OVA in PB with 0.9, 1.8 and 3.6% NaCl was also tested by high pressure size-exclusion chromatography (HP-SEC). Far-UV circular dichroism (CD) spectra of OVA before and after release were measured by using a Jasco J-815 spectropolarimeter (Tokyo, Japan). Spectra were collected from 260–190 nm, at 25°C.

To compare the *in vitro* release of OVA from AEP-MSNs and LB-MSN-OVA, OVA-loaded AEP-MSNs and LB-MSN-OVA were dispersed in PBS (pH 7.4) and incubated in the Eppendorf mixer (400 rpm, 37°C). At various time points, the suspensions were centrifuged and the supernatants were replaced with fresh PBS. The amount of OVA released into the supernatant was determined by measuring the intrinsic fluorescence intensity of OVA on a Tecan M1000 plate reader.

### Interaction of LB-MSN-OVA with Bone Marrow-Derived Dendritic Cells (BMDCs)

Dendritic cells were cultured from BALB/c donor mice as previously described (28). The study was carried out under the guidelines compiled by the animal ethic committee of the Netherlands, and approved by the ethical committee on animal experiments of Leiden University. Briefly, cell suspensions of bone marrow were obtained by flushing the femurs and tibia of adult BALB/c mice with culture medium. The cells (6 × 10^6^ cells/well) were cultured for 10 days in Iscove’s Modified Dulbecco’s Medium (IMDM) supplemented with 10% (*v*/v) fetal bovine serum, penicillin and streptomycin (100 units/ml), 20 μM beta-mercaptoethanol and 20 ng/ml GM-CSF. The cells were cultured at 37°C with 5% CO_2_. The medium was refreshed every 2 days.

To study the uptake of nanoparticles, BMDCs (2.5 × 10^5^ cells/ml) were cultured with LB-MSN-OVA containing 6 μg/ml, 0.6 μg/ml or 0 μg/ml (culture medium) OVA-AF488 for 4 h at either 4°C or 37°C. Free OVA-AF488 solution with the same concentrations was used as a control. After 4 h, the uptake of OVA-AF488 was measured using flow cytometry (FACSCanto II, Becton Dickinson, NJ). To quench the external AF488 signal, 0.02% trypan blue was added 5 min before FACS analysis. The uptake of OVA-AF488 was expressed as the mean fluorescence intensity (MFI, fluorescence intensity of each cell in average) in the AF488 channel.

To study the activation of BMDCs by the nanoparticles, BMDCs (5 × 10^5^ cells/ml) were cultured with LB-MSN-OVA containing 6 μg/ml, 0.6 μg/ml or 0 μg/ml (culture medium) OVA-AF488 for 4 h at 37°C. OVA-AF488 solution with the same concentrations and LPS (1 μg/ml) were used as controls. The cells were stained for 30 min with a mixture of 300 × diluted anti-CD40-FITC, anti-CD80-PE, and anti-CD86-APC. The cells were washed and the expression of CD40, CD80 and CD86 were quantified by flow cytometry.

### Modification of Silicon Microneedle Arrays to Obtain a pH-Sensitive Surface

To coat negatively charged particles onto silicon microneedle arrays, the microneedles were chemically modified to obtain a pH-sensitive surface (positively charged at pH 5.8) by using pyridine groups, as described previously (6). The surface of silicon was first cleaned by acetone and methanol. Next the surfaces were hydroxylated by a fresh piranha mixture consisting of 30% (*v*/v) H_2_O_2_ and 70% (*v*/v) H_2_SO_4_. Then the surface was incubated with 2% (*v*/v) APTES in toluene overnight at room temperature to obtain the amine-modified silicon surface.

The amine-modified surface was modified with 4-pyridinecarboxaldehyde (100 mM) in anhydrous isopropanol with acetic acid (1%, *v*/v) at room temperature. The obtained imine bonds on pyridine-modified surface were reduced to a secondary amine by incubating in NaBH_3_CN (50 mM) in isopropanol for 2 h. Finally the modified surface was cleaned with isopropanol and methanol and dried in a vacuum oven at 50°C for 0.5 h.

### Coating of LB-MSN-OVA on pH-Sensitive Microneedle Arrays

To determine the level of binding of LB-MSN-OVA on the microneedle arrays, DOPE-LR was added to the lipids when the LB-MSN-OVA were prepared. The top of the microneedle arrays was incubated with LB-MSN-OVA (50 μl) with a concentration of 0.1, 0.5 and 1 mg/mL in EDTA buffer (1 mM, pH 5.8) for 2 h at room temperature. The microneedles were then washed with coating buffer (450 μl) and the solution was kept for measurement. The binding efficiency of LB-MSN-OVA was determined by comparing the DOPE-LR concentration in the coating solution before and after coating by using a Tecan M1000 plate reader (Excitation wavelength = 575 nm and Emission wavelength = 590 nm). The structure, geometry and the surface morphology of the LB-MSN-OVA coated pH-sensitive microneedle arrays were examined by scanning electron microscopy (SEM) in a FEI NOVA nanoSEM 200 (Hillsboro, OR). The LB-MSN-OVA coated on microneedle arrays were also visualized by Nikon D-Eclipse C1 confocal laser scanning microscope (CLSM, Tokyo, Japan) with a depth resolution of 5 μm/step, equipped with a 10 × Plan Apo objective. The x and y resolution was 2.5 μm. An argon laser (488 nm) was used to visualize OVA-AF488 with a 530/55 emission filter and a diode-pumped solid-state laser (561 nm) with a 590/55 emission filter was used to visualize DOPE-LR.

### Delivery of LB-MSN-OVA from Microneedles into *Ex Vivo* Human Skin

After coated with LB-MSN-OVA, the pH-sensitive microneedles were pierced into human skin from the abdomen, which was used within 24 h after cosmetic surgery from a local hospital. The study was conducted in accordance to Helsinki principles and written informed patient consent was obtained. The microneedles were applied into the skin by an impact-insertion applicator with a velocity of 54.8 cm/s as described previously (6). After 1 s, the applicator was removed and the microneedles were kept inside the skin for 30 min. Then the microneedles were removed and visualized by scanning electron microscopy (SEM) in a FEI NOVA nanoSEM 200 (Hillsboro, OR). The skin was visualized by Nikon D-Eclipse C1 CLSM (Tokyo, Japan) with a depth resolution of 5 μm/step, equipped with a 4 × Plan Apo objective. The x and y resolution was 6.3 μm. An argon laser (488 nm) was used to visualize OVA-AF488 with a 530/55 emission filter and a diode-pumped solid-state laser (561 nm) with a 590/55 emission filter was used to visualize DOPE-LR.

### Statistical Analysis

All data shown are mean corrected values ± SD of at least three experiments. The results of cell experiments are analyzed by Two-way ANOVA with Bonferroni posttests.

## Results

### Characterization of MSNs and AEP-MSNs

**Fig. 1:**
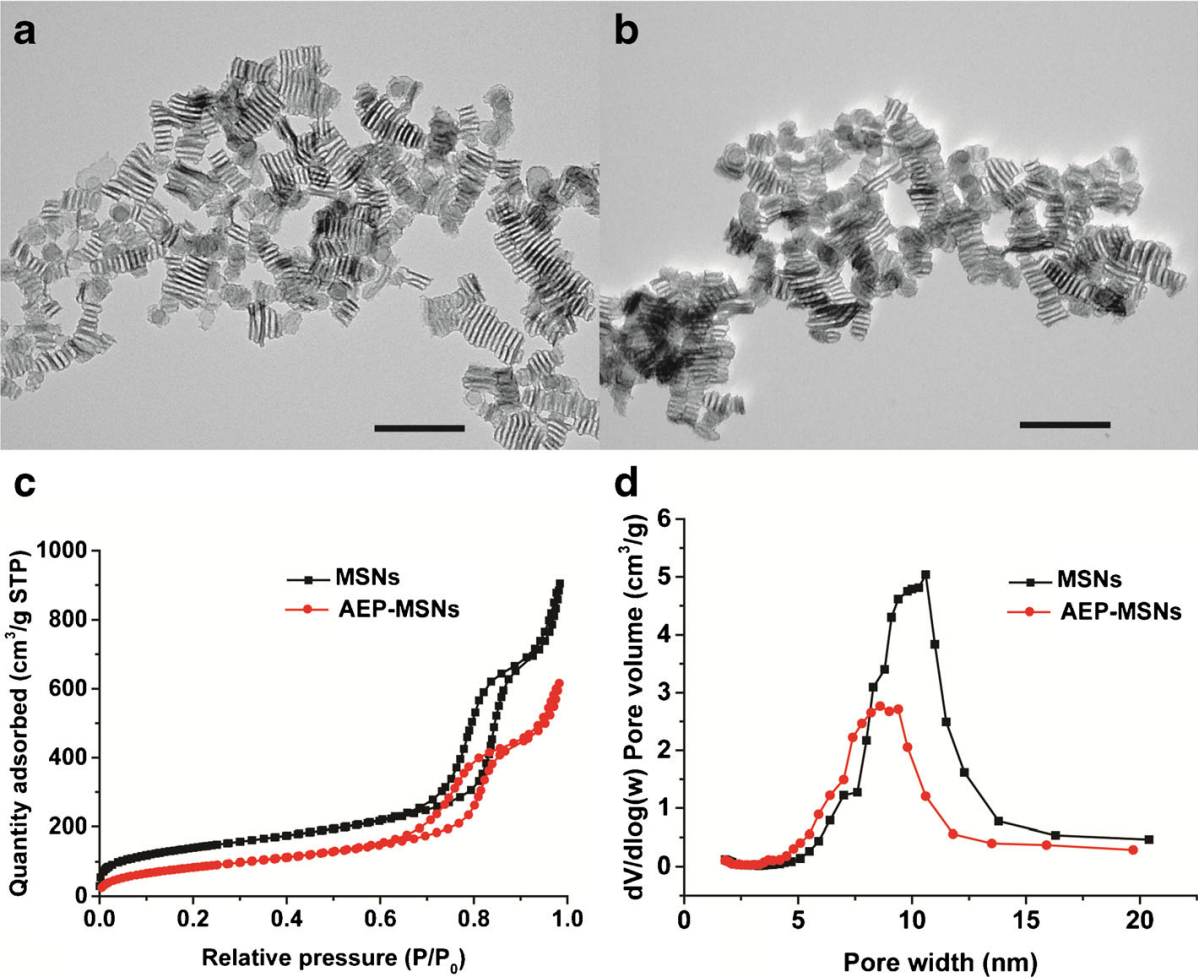
Characterization of the MSNs and AEP-MSNs. TEM images of (**a**) MSNs and (**b**) AEP-MSNs. Scale bar = 200 nm. (**c**) Nitrogen adsorption-desorption isotherms and (**d**) plots of pore diameter *vs.* pore volume (inset), calculated from the desorption isotherms using BJH model, show that the MSNs and AEP-MSNs have an average pore diameter of 10 nm and 9 nm, respectively.

The MSNs were synthesized from the silica precursor tetraethoxy silane (TEOS) by using a mixture of a nonionic triblock copolymer (Pluronic P-123) and the cationic fluorocarbon surfactant (FC-4) as organic templates. Furthermore the swelling agent TMB was added to induce the formation of large-pore MSNs (29). The obtained pristine MSNs were modified with AEPTMS in order to generate a positively charged surface (AEP-MSNs). Inspection with TEM revealed that the negatively charged MSNs were rectangular in shape with mesochannels along the short axis (Fig. 1a). Modification with AEPTMS did not alter the morphology or mesostructure (Fig. 1b), as compared to pristine MSNs. Furthermore, characterization with N_2_ adsorption-desorption isotherms of both MSNs and AEP-MSNs showed that these nanoparticles have typical IV isotherms according to International Union of Pure and Applied Chemistry (IUPAC) classification (Fig. 1c) (30). The existence of channel-type mesopores was confirmed by the existence of a type-H_1_ hysteresis loop (Fig. 1c) (31). The values for BET specific surface area (S_BET_), the total pore volume (V_t_), BJH pore diameter (W_BJH_) and surface charge of MSNs and AEP-MSNs are summarized in Table I. It can be seen that after modification with AEPTMS, S_BET_, V_t_ and W_BJH_ were slightly reduced because of the attachment of the functionalized silanes on the pore surface. The pore diameter of the AEP-MSNs was 1–2 nm smaller than that of MSNs (Fig. 1d), but still sufficiently large to accommodate OVA (4 × 5 × 7 nm) (22). Dynamic light scattering (DLS) measurements showed that the hydrodynamic diameter of MSNs and AEP-MSNs was 146.3 ± 0.3 nm and 213.7 ± 0.8 nm, respectively. The observed increase in Z-average size for AEP-MSNs may be attributed to some particle aggregation, which is probably due to the decreased charge repulsion among AEP-MSNs compared to MSNs (Table I).

**Table I:** Physical characteristics of nanoparticles (*n* = 3)

Sample	BET surface area (m^2^/g)	Pore volume (cm^3^/g)	Pore diameter (nm)^a^	Size (nm)	PDI	Zeta-potential (mV)^b^
MSNs	506	1.01	10 ± 1	146.3 ± 0.3	0.154 ± 0.035	−27.8 ± 0.4
AEP-MSNs	318	0.71	9 ± 1	213.7 ± 0.8	0.170 ± 0.062	10.9 ± 0.5
AEP-MSN-OVA	-	-	-	1842 ± 126	0.373 ± 0.056	−8.1 ± 1.3
LB-MSN-OVA	-	-	-	190.7 ± 2.7	0.125 ± 0.029	−24.0 ± 0.7

^a^Calculated from desorption branch of the N_2_ sorption isotherms based on the BJH method

^b^Zeta-potential was measured in 1 mM PB at pH 7.4

### Encapsulation and Release of OVA from AEP-MSNs

**Fig. 2:**
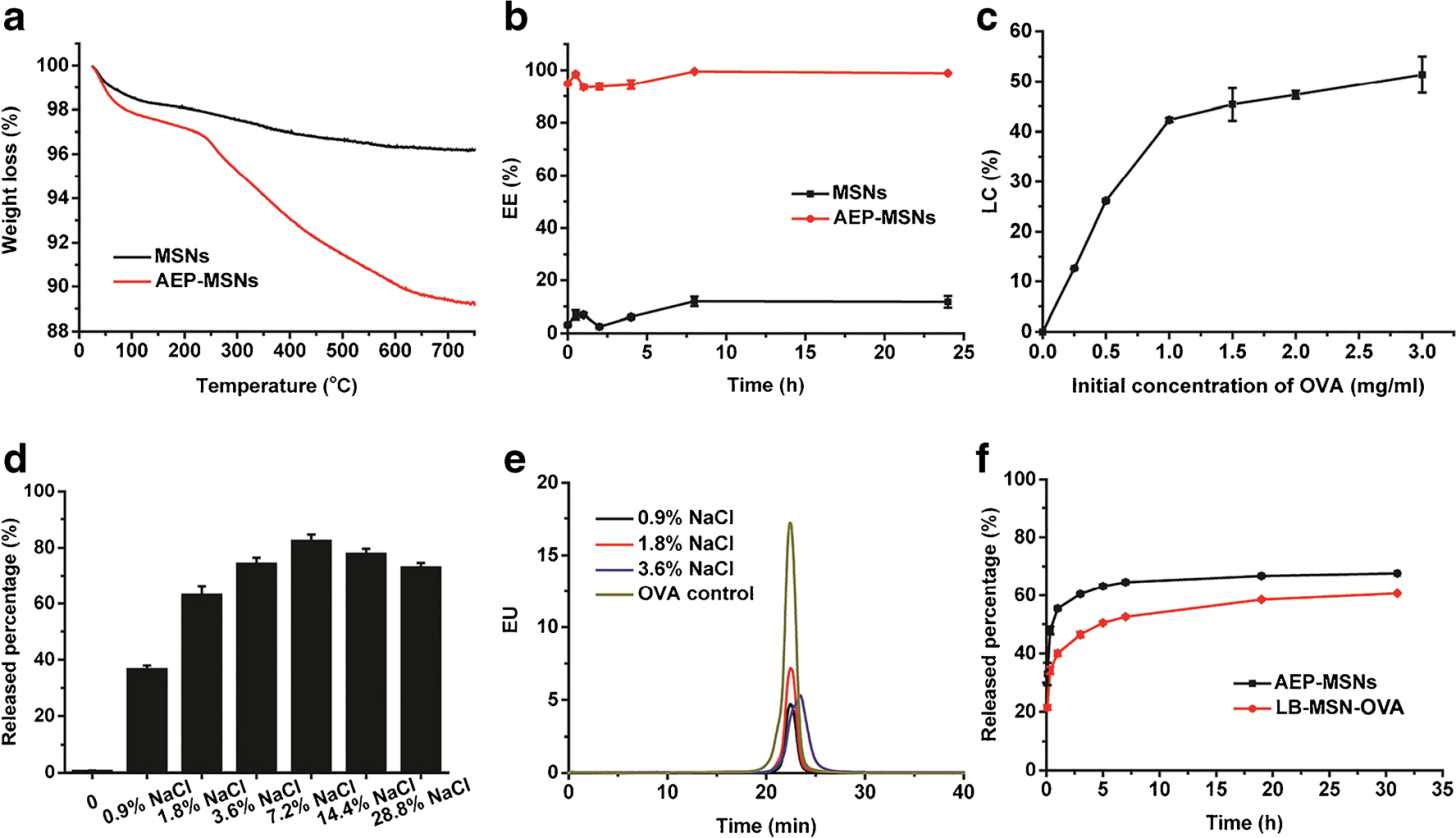
(**a**) Thermogravimetric analysis (TGA) curves of MSNs and AEP-MSNs. (**b**) Encapsulation kinetics of OVA into MSNs and AEP-MSNs (mean ± SD, *n* = 3), concentration of OVA is 0.5 mg/mL and MSNs (AEP-MSNs) is 2 mg/mL. (**c**) Loading capacity (LC%) of OVA into AEP-MSNs (mean ± SD, *n* = 3) at different initial concentration of OVA. (**d**) Influence of ionic strength on OVA release from AEP-MSNs (mean ± SD, *n* = 3). (**e**) HP-SEC chromatograms of the released OVA from AEP-MSNs. (**f**) Release profiles of OVA from AEP-MSNs and LB-MSN-OVA in PBS (pH 7.4) (mean ± SD, *n* = 3).

The percentage of grafted amine-containing groups on the surface of AEP-MSNs was 6.9%, as determined by thermogravimetric analysis (TGA, see Fig. 2a). The encapsulation efficiency (EE%), defined as the percentage of OVA which is adsorbed in the MSNs or AEP-MSNs was determined as a function of incubation time (Fig. 2b). The calibration curve used to calculate the concentration of OVA is shown in supplementary Fig. 1a. This study revealed that the OVA encapsulation within AEP-MSNs was very efficient, as 95 ± 0.4% (mean ± SD, *n* = 3) of the protein was encapsulated in the AEP-MSNs. Furthermore, equilibrium of OVA encapsulation was reached in less than 5 min. In comparison, only 12 ± 2% (mean ± SD, *n* = 3) of OVA was encapsulated in negatively charged MSNs after 24 h. The loading capacity (LC%) of OVA was calculated from the amount of OVA encapsulated in AEP-MSNs and expressed as the percentage of the total weight of OVA-loaded AEP-MSNs. The LC% of OVA in AEP-MSNs was dependent on the initial concentration of OVA (Fig. 2c). The maximum LC% was 34 ± 4% (mean ± SD, *n* = 3) was achieved by increasing the initial concentration of OVA, indicating a diffusion-driven encapsulation process (32).

To examine the influence of ionic strength of the medium on the release profile of OVA from the AEP-MSNs, the concentration of NaCl in the buffer was varied. The calibration curve used to calculate the concentration of OVA is shown in supplementary Fig. 1b. The release percentage of OVA (defined as the percentage of OVA released from total encapsulated OVA in AEP-MSNs) increased from 0.6 ± 0.2% (mean ± SD, *n* = 3) in NaCl-free buffer to 82 ± 2% (mean ± SD, *n* = 3) in buffer containing 7.2% NaCl (Fig. 2d). These results demonstrate that the ionic strength of the medium plays an important role in the release of OVA, indicating that the interaction between OVA and AEP-MSNs is mainly electrostatic in nature. The structural integrity of the released OVA was examined by HP-SEC, showing that the released OVA was mainly monomeric (Fig. 2e), and far-UV CD spectroscopy, indicating that the secondary structure of released protein was similar to that of native OVA (supplementary Fig. 2). These results strongly indicate that encapsulation and release have no adverse effect on the protein structure.

### Preparation and Characterization of LB-MSN-OVA

**Fig. 3:**
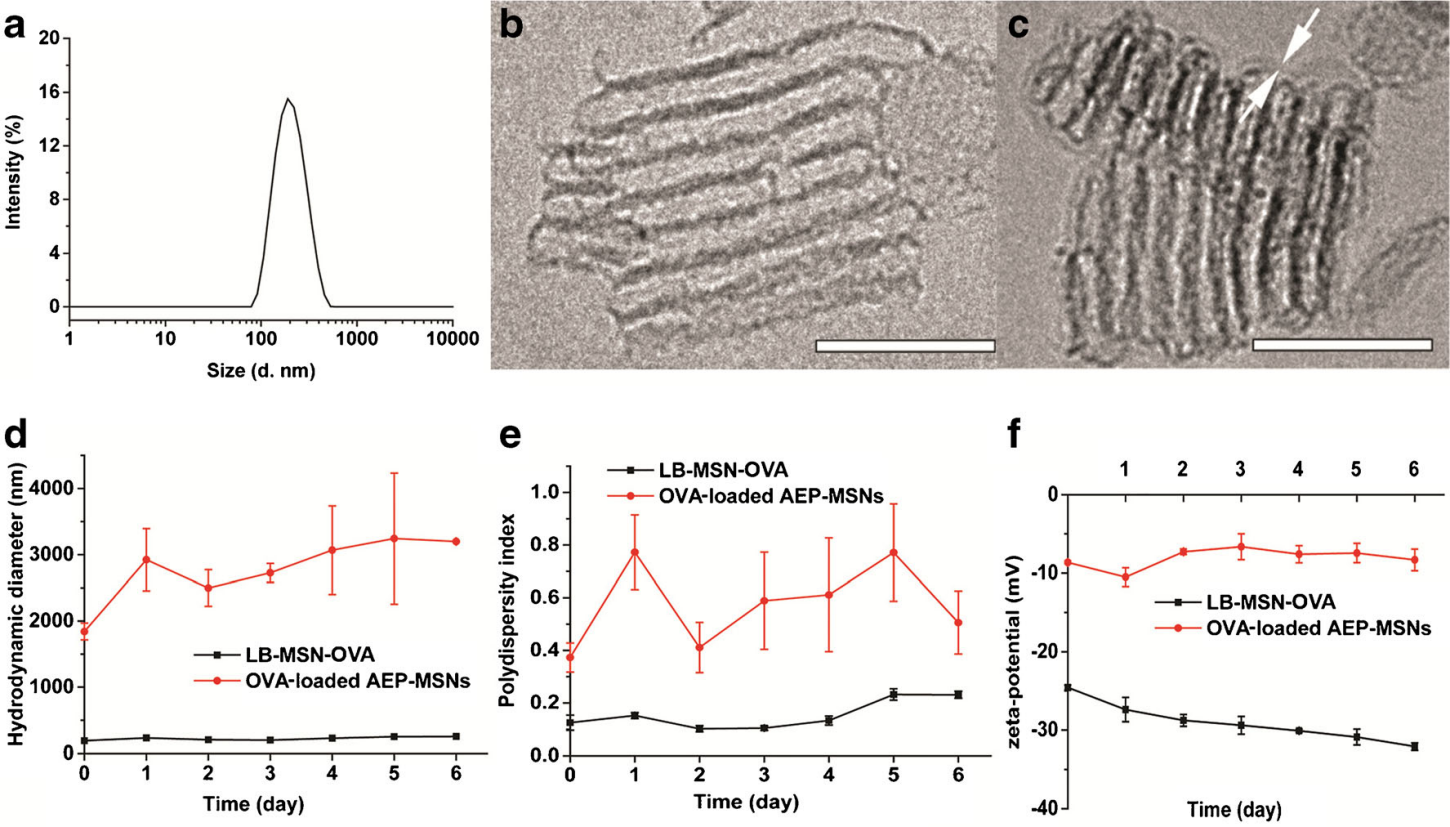
Characterization of LB-MSN-OVA. (**a**) Hydrodynamic diameter of LB-MSN-OVA determined by DLS. (**b**) CryoTEM image of AEP-MSNs, and (**c**) LB-MSN-OVA, revealing a lipid bilayer thickness of ~4 nm (indicated by white arrows), scale bar = 100 nm. (d-f) colloidal stability of OVA-loaded AEP-MSNs and LB-MSN-OVA over one week: (**d**) hydrodynamic diameter, (**e**) polydispersity index and (**f**) zeta potential).

The OVA-loaded AEP-MSNs had the tendency to precipitate and form large aggregates (Table I), probably due to the decreased surface charge upon protein encapsulation (−8.1 ± 1.3 mV, mean ± SD, *n* = 3). In order to increase the colloidal stability, the OVA-loaded AEP-MSNs were stabilized with a lipid bilayer composed of DOPC, DOPS and cholesterol. For this, liposomes and OVA-loaded AEP-MSNs were mixed and equilibrated for 1.5 h and afterwards the excess lipids were removed by centrifugation. The encapsulation efficiency of OVA in the resulting lipid-coated AEP-MSNs (LB-MSN-OVA) was determined to be 74 ± 1%, as compared to 99 ± 1% without lipid (mean ± SD, *n* = 3). The obtained LB-MSN-OVA were characterized by DLS, NTA and TEM. The mean number-based hydrodynamic diameter (176 ± 11 nm, mean ± SD, *n* = 3) measured by NTA (supplementary Fig. 3) was close to the Z-average hydrodynamic diameter (190.7 ± 2.7 nm; PDI = 0.125 ± 0.029; mean ± SD, *n* = 3) found by DLS (Fig. 3a). The existence of a lipid bilayer surrounding the AEP-MSNs was confirmed by cryoTEM (Fig. 3b and c). The colloidal stability of the formulation was examined by measuring the hydrodynamic diameter and zeta-potential of LB-MSN-OVA for one week (Fig. 3d-f). It showed that LB-MSN-OVA slightly changed in diameter and zeta-potential revealing that the lipid bilayer strongly enhanced the colloidal stability. The release of OVA from AEP-MSNs and LB-MSN-OVA was examined in PBS (pH 7.4) for 32 h (Fig. 2f). The burst release of OVA from LB-MSN-OVA was less in comparison to AEP-MSNs, indicating that the lipid bilayer acts as a barrier retaining the OVA for longer inside the AEP-MSNs.

### Interaction of LB-MSN-OVA with BMDCs

**Fig. 4:**
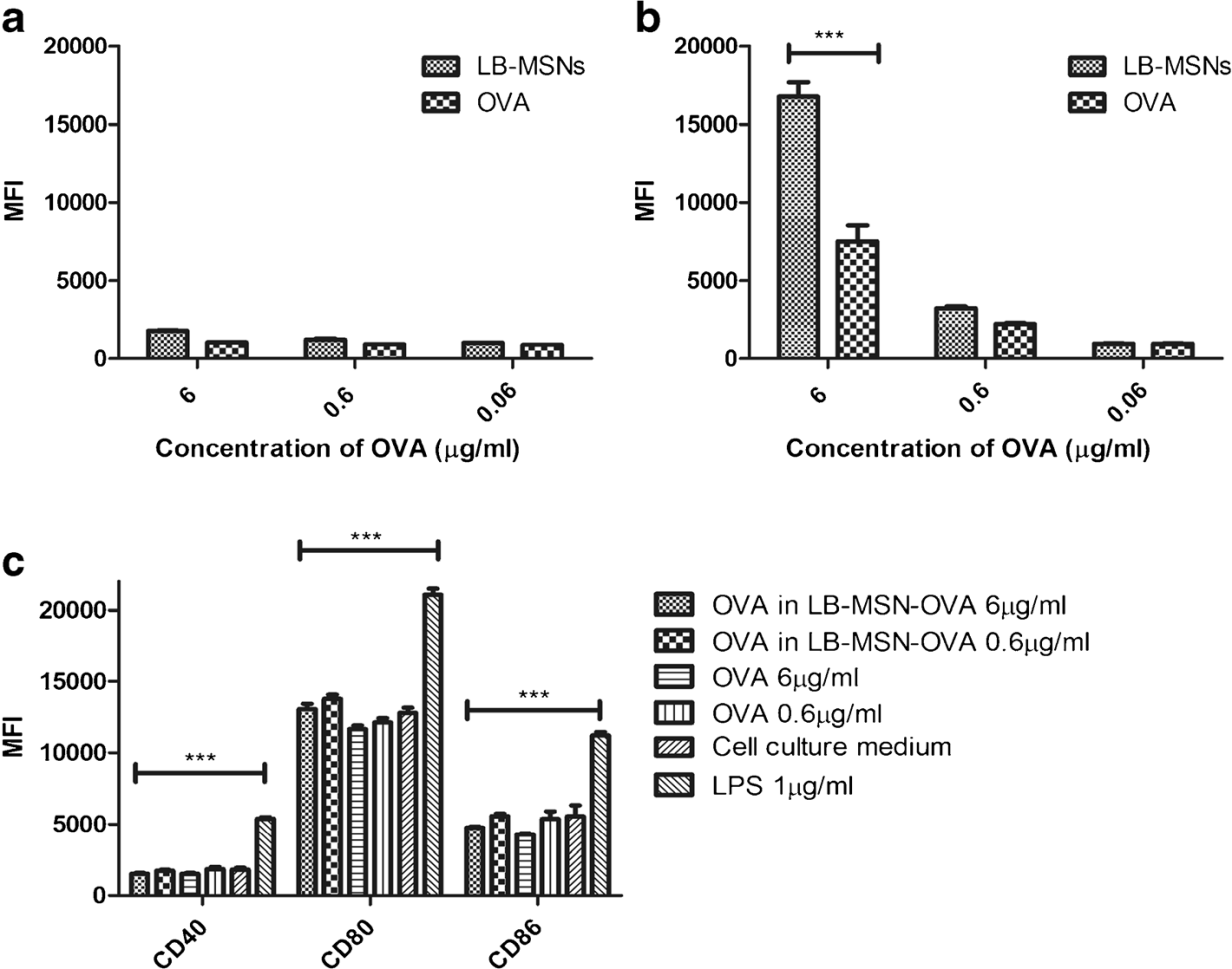
The uptake of LB-MSN-OVA in BMDCs at 4°C (**a**) and 37°C (**b**), and the activation of BMDCs by LB-MSN-OVA (**c**). Bars represent mean ± SD, *n* = 3. The uptake of OVA-AF488 and expression of CD40, CD80 and CD86 were expressed as the mean fluorescence intensity (MFI). *** P<0.001.

As proteins in serum may interact with the particles, the colloidal stability of LB-MSN-OVA in cell culture medium was studied. Only limited aggregation of the nanoparticles was observed and a modest amount of OVA (15%) was released after 4 h (supplementary Table 1). To examine whether LB-MSN-OVA facilitate the uptake by BMDCs, the uptake of LB-MSN-OVA was assessed by flow cytometry and compared to that of free OVA solution. As shown in Fig. 4, at 4°C there was almost no uptake (no significance compared to culture medium only) of LB-MSN-OVA or OVA in BMDCs (Fig. 4a), indicating that the uptake of LB-MSN-OVA and OVA is mediated by an active process. At 37°C the fluorescent level of LB-MSN-OVA treated cells was significantly higher (P<0.001) than that for free OVA-AF488 with the OVA concentration of 6 μg/ml (Fig. 4b). There was no significant difference found between LB-MSN-OVA and free OVA at lower concentration. These results indicate that LB-MSN-OVA are capable of promoting antigen uptake by antigen-presenting cells (BMDCs). In order to study the activation of BMDCs by the nanoparticles, BMDCs were incubated with different formulations for 4 h and the expression of CD40, CD80 and CD86 was measured. Whereas exposure to LPS led to a significant upregulation of these activation markers, LB-MSN-OVA did not induce increased expression of CD40, CD80 or CD86 on dendritic cells compared to free OVA or cell culture medium (Fig. 4c).

### Coating of LB-MSN-OVA on Microneedles

Next, we investigated whether the LB-MSN-OVA could be adsorbed onto a silicon microneedle array via physical adsorption. First, the pH-sensitive pyridine-modified microneedle arrays were prepared as described previously (6). The microneedle arrays were coated with LB-MSN-OVA at pH 5.8 in an EDTA buffer (1 mM). To determine the optimal concentration of LB-MSN-OVA for the coating process, the nanoparticle concentration was varied in the buffered coating solution. Increasing the LB-MSN-OVA concentration resulted in increased amounts of LB-MSN-OVA coated onto the microneedle array surfaces. However, the coating efficiency is reduced (Table II). The lowest coating efficiency obtained was 16 ± 2.7% (mean ± SD, *n* = 3), corresponding to 7.9 ± 1.3 μg (mean ± SD, *n* = 3) and 1.5 ± 0.24 μg (mean ± SD, *n* = 3) of LB-MSN-OVA and OVA, respectively coated on the microneedle array. Considering the surface area of the microneedles accounts for 40% of the total surface area of microneedle arrays, 3.2 ± 0.5 μg (mean ± SD, *n* = 3) of nanoparticles and 0.58 ± 0.10 μg (mean ± SD, *n* = 3) of OVA were coated onto the microneedle surface of one array.

**Table II:** Coating amount of LB-MSN-OVA and OVA on microneedle arrays

Amount of LB-MSN-OVA^a^ (μg)	Coated LB-MSN-OVA (μg)	Coated OVA^b^ (μg)	Coating efficiency (%)
5	1.3 ± 0.2	0.24 ± 0.03	27 ± 3
25	5.4 ± 1.7	1.0 ± 0.3	22 ± 7
50	7.9 ± 1.3	1.5 ± 0.2	16 ± 3

^a^The amount of LB-MSN-OVA in coating solution; ^b^The amount of coated OVA was calculated from the loading capacity of OVA and the coating amount of LB-MSN-OVA. All the coating amounts are expressed as the amount of AEP-MSNs and are based on one microneedle array which contains 576 needles per array. All the results are based on 3 independent microneedle arrays

**Fig. 5:**
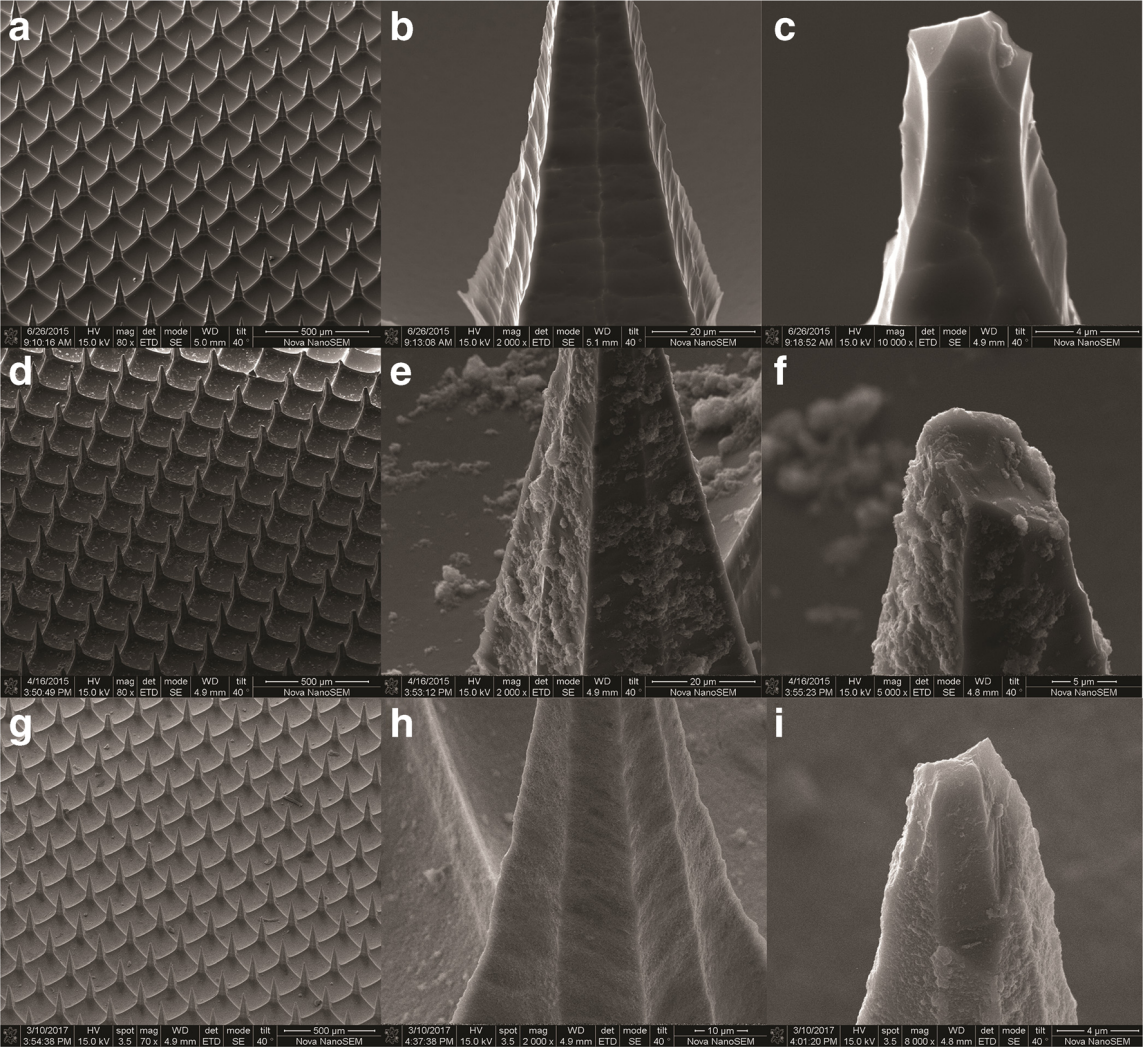
SEM images of pyridine-modified microneedle arrays before the adsorption of LB-MSN-OVA with different magnifications (a: 80 ×; b: 2000 ×; c: 5000 ×), after the adsorption of LB-MSN-OVA with different magnifications (d: 80 ×; e: 2000 ×; f: 5000 ×) and after the penetration of human skin (g: 80 ×; h: 2000 ×; i: 5000 ×).

**Fig. 6:**
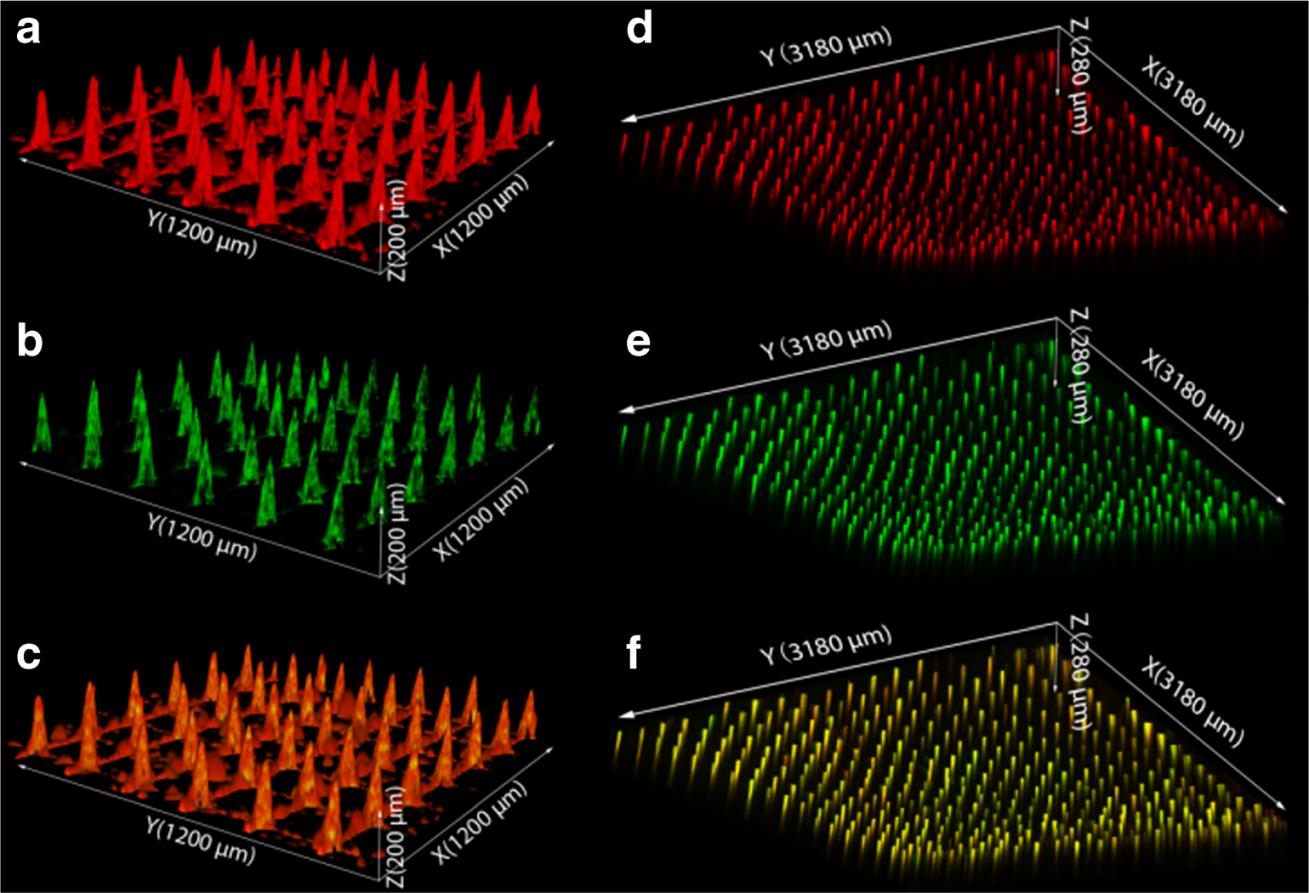
CLSM images of LB-MSN-OVA coated microneedles (a-c). Red: DOPE-LR (**a**); Green: OVA-AF488 (**b**); Merged (**c**). The x and y arrows show that the scanning area is 1200 μm × 1200 μm large. The z arrow indicates the scanning depth of 200 μm. CLSM images of human skin after removal of the LB-MSN-OVA coated microneedle arrays (d-f). Red: DOPE-LR (**d**); Green: OVA-AF488 (**e**); Merged (**f**). The x and y arrows show that the scanning area is 3180 μm × 3180 μm large. The z arrow indicates the scanning depth of 280 μm.

Scanning electron microscopy imaging was used to visualize the presence of the LB-MSN-OVA on the pyridine-modified microneedle arrays (Fig. 5a-f). Compared to untreated pyridine-modified arrays (Fig. 5a-c), a high number of nanoparticles were observed on the surface of the microneedles (Fig. 5d-f) after coating with LB-MSN-OVA. To determine whether the OVA and nanoparticles colocalized on the microneedles, the LB-MSN-OVA coated microneedles were visualized by CLSM. For this experiment, we used OVA-AF488 and DOPE-LR enabling the visualization of both the protein and lipids. Imaging revealed that the fluorescent labels were both located at the microneedle surfaces indicative of the integrity of the LB-MSN-OVA upon physical adsorption (Fig. 6a-c). This showed us that LB-MSN-OVA could be immobilized onto microneedles via electrostatic interaction.

### Delivery of LB-MSN-OVA into Human Skin

Next, the delivery of LB-MSN-OVA from the surface of microneedles into the skin was studied. For this, the nanoparticle-coated microneedle arrays were applied onto human skin *ex vivo* for 30 min and subsequently withdrawn. Next the intradermal delivery was studied by both SEM and CLSM. Less particles were observed on surface of microneedles after the penetration and withdrawal from human skin (Fig. 5g-i). Colocalization of the fluorescence from both OVA-AF488 and DOPE-LR was observed inside the skin (Fig. 6d-f), illustrating that the microneedles penetrated into the skin and successfully delivered the LB-MSN-OVA.

## Discussion

An alarming trend towards decreased vaccine compliance in the western world emphasizes the need to develop effective, but also safe and easily administrable vaccines. In this respect dermal vaccination is interesting as the skin provides an easily accessible (and potentially painless) route of administration and also provides an environment which is very conductive for the initiation of immunological memory. Topical administration of vaccines is often not effective as bulky vaccines do not permeate the skin. Recently, we and other groups have shown that antigens can effectively be delivered into the epidermis and dermis by means of coated microneedles (3,4,10,33). However, some major challenges remain, which include the effective dose that can be delivered with coated microneedles and the immunogenicity of the subunit vaccines (4,6).

Here we introduce a novel carrier system for subunit vaccines with a high loading efficiency that effectively delivers a model antigen into the skin using a complementary charged microneedle array. To our best knowledge, the current study is the first example of a microneedle-mediated intradermal delivery system for mesoporous nanoparticles, which could be a promising tool to deliver a wide range of compounds into the skin. High loading efficiency was achieved by encapsulating the model antigen OVA into surface-modified MSNs with large pores (>10 nm). We chose MSNs because of their advantageous properties, including large surface area, controlled particle size and pore structure as well as ease of surface modification. Moreover, a previous study showed that subcutaneous immunization with 2 μg of OVA-loaded MSNs induced comparable antibody responses as 50 μg OVA adjuvanted with Quil-A (18), demonstrating that antigen-loaded MSNs can elicit an immune response at reduced antigen doses compared to a conventional delivery system. Our results indicate that one of the reasons for the immune enhancing effect on MSNs may be the increased uptake by dendritic cells when OVA is associated with MSNs (Fig. 4). LB-MSN-OVA do not increase the activation of dendritic cells compared to free OVA, which is in line with previous findings (34). Similar results were also reported with OVA-loaded PLGA nanoparticles (35) as OVA-loaded PLGA nanoparticles were found not to increase activation of human monocyte-derived dendritic cells (MHC II, CD83 and CD86). This suggests that the addition of adjuvants capable of inducing DC maturation, may further increase the immunogenicity of LB-MSN-OVA.

For an efficient dermal delivery of nanoparticulate vaccines, MSNs are required that are small in size. In addition, they should have large pores (inner diameter > 5 nm) in order to encapsulate large amounts of proteins. Most nanosized MSNs do not fit these criteria, although recently some examples have emerged, mainly for the delivery of DNA/RNA (24,36–39). MSNs with a large pore size of about 10 nm, recently developed in our lab (26), were used in the current study to accommodate the relatively large OVA molecules (4 × 5 × 7 nm). The encapsulation study showed that the synthesized MSNs can accommodate a large amount of OVA within 5 min after mixing AEP-MSNs with OVA. It has been reported that MSNs with a pore size of 3.6 and 2.3 nm had a maximum OVA LC% of 21.8% (1) and 7.2% (18), respectively. The even higher maximum LC% of OVA in our study of 33.9% may be due to the larger pore size.

To coat nanoparticles onto the pyridine-modified microneedles, the nanoparticles should have a negative surface charge allowing for adsorption based on electrostatic interactions, and a good colloidal stability allowing uniform and reproducible coating. In our study, negative liposomes were used to fuse to the surface of the positively charged AEP-MSNs, to achieve a negative surface charge. This fusion method was previously used for coating fluorophore (40), photosensitizers (41) and DNA loaded MSNs (23) and was reported to be based on the electrostatic interaction between the lipids and surface of MSNs (23). The fusion of lipid bilayer on MSN surface has been shown to be able to modify the charge, improve the stability of MSNs and contain the drug inside the pores of MSNs. In order to prepare the liposomes, DOPC and cholesterol were used because in a previous study liposomes containing DOPC and cholesterol were shown to be able to stabilize drug-, small interfering RNA- and toxin-loaded MSNs (25). DOPS was used to give the liposomes a negative charge, which is needed to coat the nanoparticles onto the positively charged microneedles. Our results show that the colloidal stability of OVA-loaded MSNs was improved after liposome fusion and the lipid bilayer generated a negatively charged surface on LB-MSN-OVA. The LB-MSN-OVA were coated onto microneedles at pH 5.8 where more than 90% of the pyridine groups are positively charged (6). Combined with the low ionic strength of the buffer, this allows for the binding of the negatively charged LB-MSN-OVA via electrostatic interactions. The presence of the lipid bilayer on the surface of MSNs was confirmed by cryoTEM and indicated by the change of surface charge (from +11 mV to −24.0 mV at pH 7.4). The encapsulation efficiency of OVA was decreased by about 25% after the fusion of liposomes, which may be because the negatively charged lipid bilayer and OVA were competing with each other for the binding on the MSN surface and some of the OVA coated on the AEP-MSN surface may be replaced by the lipid bilayer. The release study showed that the coated lipid bilayer functioned as a gate and prolonged the release of the antigen, which could be important for the nanoparticles to remain their adjuvant effect (35).

The binding of LB-MSN-OVA on microneedles was visualized by both SEM and CLSM. The SEM images showed that after coating the microneedles with LB-MSN-OVA, the surface of the microneedles became rougher, but the sharpness of the microneedles was not affected. One major disadvantage of coated microneedles is the limited amount of materials that can be coated on microneedles because of the small surface area. The amount of LB-MSN-OVA coated on one microneedle array was 7.9 μg and was higher than that of inactivated polio virus (IPV) in a previous study (100 ng) (33). Thus next to improving the immunogenicity of antigens, LB-MSN-OVA could also provide an effective way of increasing the antigen dose coated on microneedles. This may be because LB-MSN-OVA have a lower zeta potential than IPV under similar conditions (−16.8 mV vs − 7.8 mV in 1 mM EDTA at pH 5.8). In our study the coated OVA loaded in LB-MSN-OVA is 1.5 μg on one microneedle array and is much higher than the amount of coated IPV (33) in a previous study. Other possibilities to increase the delivered amount of antigen are increasing the number of microneedle arrays used or increasing the number of needles on one array.

To effectively deliver antigens into the skin, next to efficient coating of the antigen on the microneedles, rapid dissolution from the microneedles once inserted into the skin, is critical. The pH-sensitive microneedles used in the present study were developed in our lab for the intradermal delivery of vaccines by coating antigens at slightly acidic pH and releasing them at physiological pH. CLSM images showed that the LB-MSN-OVA were successfully released into the holes made by the microneedles. The fluorescence from lipids and OVA was found to still co-localize with each other in the holes made by microneedles, indicating that the LB-MSN-OVA may be still intact after the release. This would be important for LB-MSN-OVA to remain their adjuvant effect (25).

Thus, the developed system combines the advantages of microneedles and nanoparticles. Microneedles allow non-invasive delivery of vaccines into skin and antigen-loaded nanoparticles have the potential to increase and modify the immune response against the antigen. In addition, by coating the nanoparticles onto the pH-sensitive pyridine-modified microneedles, the separate application of antigen after microneedle penetration is avoided. An important concern is the bio-distribution of MSNs after intradermal delivery. Studies have shown that intravenously injected MSNs were mainly excreted out of mice through urine and feces, indicating that MSNs are biodegradable (42) and other studies showed that MSNs can undergo hydrolysis to form non-toxic silica acid (43). However, as deposition in the skin may alter the biodistribution and clearance of the MSNs, systematic studies need to be performed in order to assess the safety of these nanoparticles in animals and humans.

## Conclusion

In conclusion, the LB-MSN-OVA coated microneedle arrays represent a novel intradermal antigen delivery system. The large pores of MSNs enabled the rapid encapsulation of OVA with a high loading capacity. The introduction of lipid bilayers significantly improved the colloidal stability of OVA-loaded AEP-MSNs and concomitantly reduced the premature release of OVA. In addition, it enabled the coating of the nanoparticles on the surface of pH-sensitive microneedle arrays. Application of LB-MSN-OVA coated microneedle arrays into human skin (*ex vivo*) resulted in the successful delivery of the OVA-loaded nanoparticles into the skin. The method is not restricted to the delivery of antigens, but may also be useful to deliver any compound that can be encapsulated in MSNs like (low-molecular-weight) drugs, RNA, DNA and proteins.

### Acknowledgments and Disclosures

Jing Tu and Guangsheng Du acknowledge the support from the Chinese Scholarship Council. We acknowledge Pim Schipper for technical assistance with the pyridine modification of the silicon surface. Aimee Boyle is thanked for the critical reading of this manuscript. Romain Leboux and Naomi Benne are thanked for dendritic cell studies. Alexander V. Korobko helped with BET measurements at Delft University of Technology. The microneedle arrays are gift from Michael Stumber (Robert Bosch GmbH).

## Electronic supplementary material

ESM 1 (DOC 138 kb)

## Abbreviations

AEP-MSNs: AEPTMS-modified MSNs
AEPTMS: 3-[2-(2-aminoethylamino)ethylamino] propyltrimethoxysilane
BDMCs: Bone marrow-derived dendritic cells
DOPC: 1,2-dioleoyl-sn-glycero-3-phosphocholine
DOPE-LR: 1,2-dioleoyl-sn-glycero-3-phosphoethanolamine-N-(lissamine rhodamine B sulfonyl) (ammonium salt)
DOPS: 1,2-dioleoyl-sn-glycero-3-[phospho-_L_-serine](sodium salt)
EE: Encapsulation efficiency
LB-MSN-OVA: Lipid bilayer-coated and antigen-loaded AEP-MSNs
LC: Loading capacity
MSNs: Mesoporous silica nanoparticles
OVA: Ovalbumin
OVA-AF488: Alexa Fluor®488 ovalbumin conjugates
