# Supplementary material for: Mesoporous Silica Nanoparticle-Coated Microneedle Arrays for Intradermal Antigen Delivery
Source: Pharm Res. 2017 May 23;34(8):1693–706. doi: 10.1007/s11095-017-2177-4 (PMC5498618; doi:10.1007/s11095-017-2177-4)
Supplement: Supplementary file 1 — (DOC 138 kb) [file 11095_2017_2177_MOESM1_ESM.doc]

Mesoporous Silica Nanoparticle-Coated Microneedle Arrays for Intradermal Antigen Delivery

Jing Tu ^1,†^ **∙** Guangsheng Du ^2,†^ **∙** M. Reza Nejadnik ^2^ **∙** Juha Mönkäre ^2^ **∙** Koen van der Maaden ^2^ **∙** Paul H. H. Bomans ^3^ **∙**  Nico A. J. M. Sommerdijk ^3^ **∙**  Bram Slütter ^2,4^ **∙** Wim Jiskoot  ^2^  **∙** Joke A. Bouwstra ^2,*^ **∙** Alexander Kros ^1,*^

^1^ Department of Supramolecular & Biomaterials Chemistry, Leiden Institute of Chemistry (LIC), Leiden University, Leiden, 2300 RA, The Netherlands

^2^ Division of Drug Delivery Technology, Cluster BioTherapeutics, Leiden Academic Centre for Drug Research (LACDR), Leiden University, Leiden, 2300 RA, The Netherlands

^3^ Laboratory of Materials and Interface Chemistry & Center of Multiscale Electron Microscopy, Department of Chemical Engineering and Chemistry, and Institute for Complex Molecular Systems, Eindhoven University of Technology, Eindhoven, 5600 MB, The Netherlands

^4^ Division of Biopharmaceutics, Cluster BioTherapeutics, Leiden Academic Centre for Drug Research (LACDR), Leiden University, Leiden, 2300 RA, The Netherlands

† These authors contributed equally.

Supplementary Information


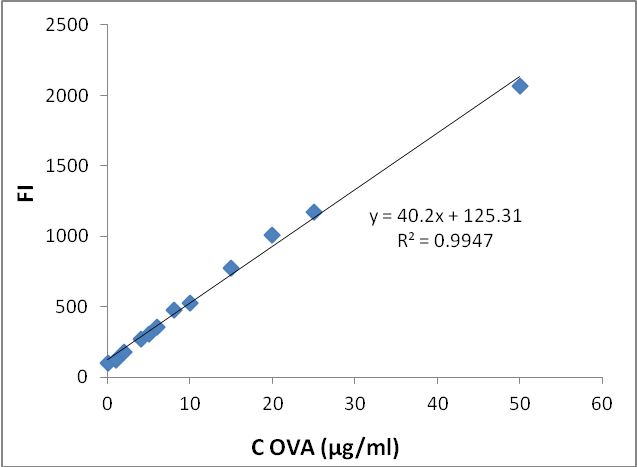

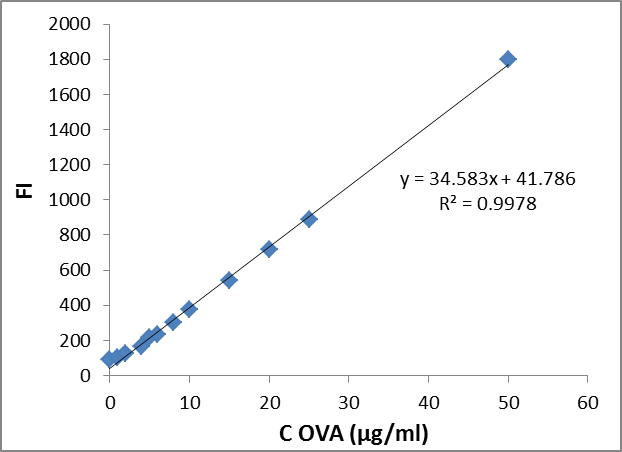


**a**

**b**

**Supplementary Fig. 1** Calibration curves for quantification of OVA in 1 mM PB with a pH of 7.4 (a) and PBS with a pH of 7.4 (b). The intrinsic fluorescence intensity (FI) of OVA was measured with an excitation wavelength of 280 nm and an emission wavelength of 320 nm.


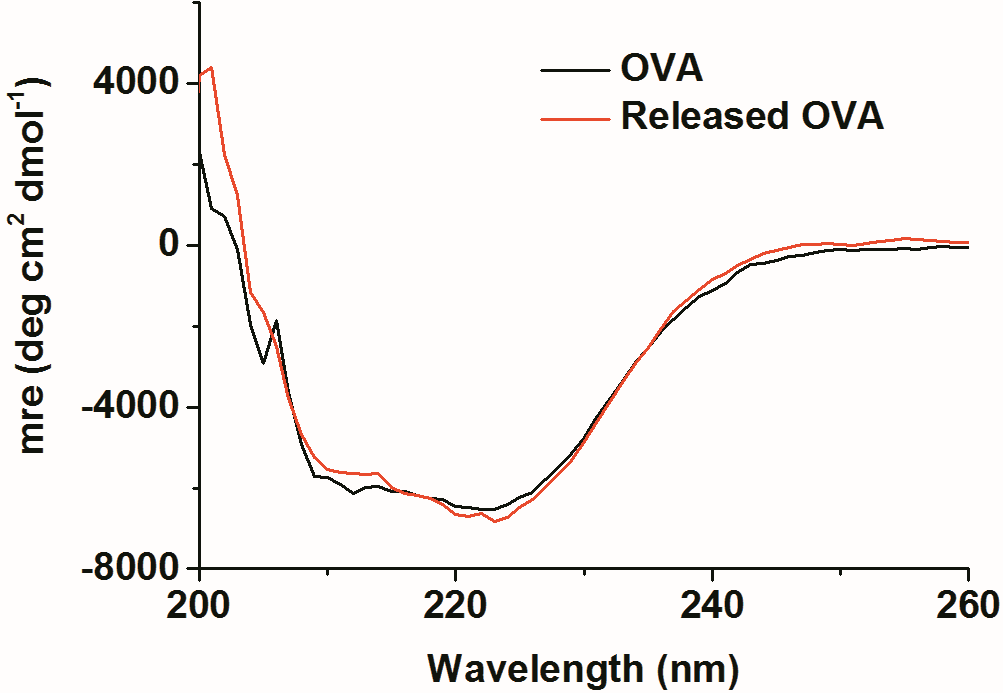


**Supplementary Fig. 2** Far-UV CD spectra of free OVA and OVA released from AEP-MSNs in PBS, pH 7.4, 25 °C.


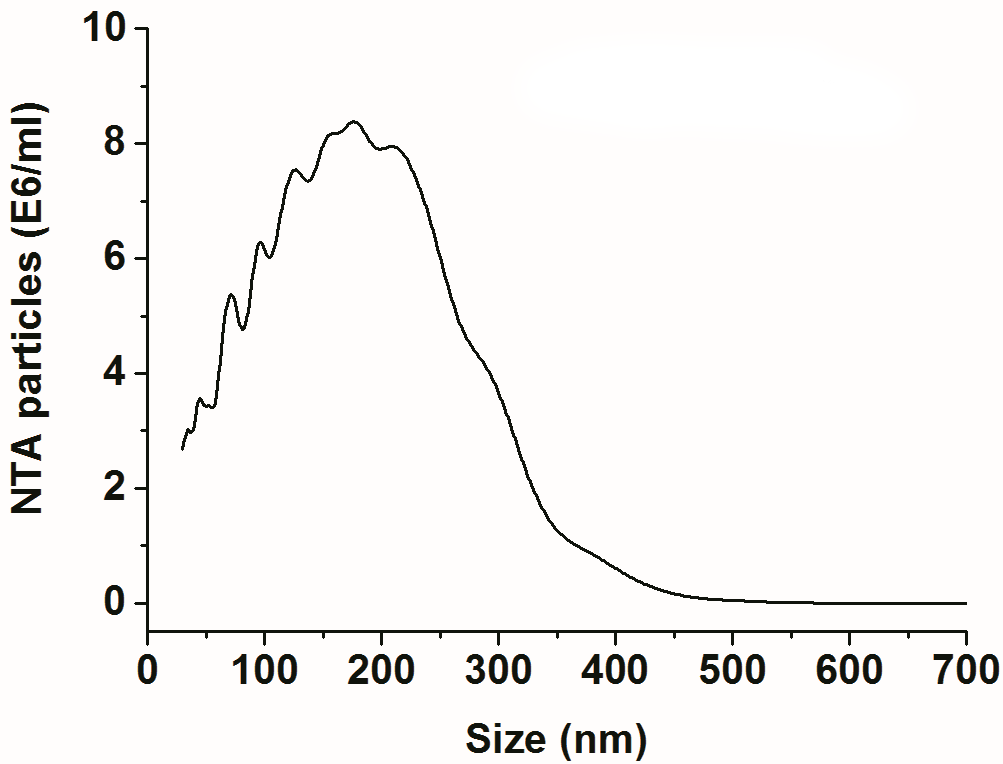


**Supplementary Fig. 3** Size distribution of the LB-MSN-OVA determined by nanoparticle tracking analysis (NTA).

**Supplementary Table 1.** Stability of LB-MSN-OVA in cell culture medium (n=3).

| Time (h) | Size (nm) | PDI | Zeta Potential (mv) | Released OVA (%) |
| --- | --- | --- | --- | --- |
| 0 | 632.5 ± 13.9 | 0.528 ± 0.031 | -10.8 ± 0.6 |  |
| 1 | 575.0 ± 18.3 | 0.535 ± 0.051 | -11.7 ± 0.4 |  |
| 2 | 536.5 ± 19.6 | 0.584 ± 0.057 | -12.8 ± 0.5 |  |
| 4 | 566.1 ± 64.5 | 0.485 ± 0.158 | -13.3 ± 0.2 | 15.2 ± 0.6 |

**Calculation of OVA loading assuming a monolayer on a solid silica sphere**

**References:**

Density of silica: 2.65g/cm^3^ (*Wikipedia,* [*https://en.wikipedia.org/wiki/Silicon_dioxide*](https://en.wikipedia.org/wiki/Silicon_dioxide) )

Dimeter of silica nanoparticles: 213 nm (*manuscript*)

Size of OVA molecule: 4 × 5 × 7 nm (*Hudson S, Cooney J, Magner E. Proteins in mesoporous silicates. Angewandte Chemie. 2008;47(45):8582-8594*)

Molecular weight of OVA: 44.3 KDa (*Sigma-aldrich,* [*http://www.sigmaaldrich.com/content/dam/sigma-aldrich/docs/Sigma/Product_Information_Sheet/a5503pis.pdf*](http://www.sigmaaldrich.com/content/dam/sigma-aldrich/docs/Sigma/Product_Information_Sheet/a5503pis.pdf))

**Calculation:**

1) The volume of 1g silica= 1g/2.65g/cm^3^=0.38 cm^3^

2) Ratio between surface area and volume of silica particles with a diameter of 213 nm= 4∏r^2^/(4/3∏r^3^)=3/r=3/(213/2 nm)=2.82 × 10^5^ cm^-1^

3) Surface area of 1g silica sphere= 0.38 cm^3^ × 2.82 × 10^5^ cm^-1^ = 1.07 × 10^5^ cm^2^

4) Surface area of single OVA molecule on surface of silica sphere = 4 nm × 5 nm = 2.0 × 10^-13^ cm^2^

Calculation is based on the assumption that OVA lies on surface with shortest side in a tight and ordered way

5) Mole amount of OVA on surface of 1g silica sphere = 1.07 × 10^5^ cm^2^/2.0 × 10^-13^ cm^2^/ 6.02 × 10^23^=8.89× 10^-7^ Mol

6) Mass of OVA molecule on surface of 1g silica sphere = 8.89× 10^-7^ Mol × 44.3 KDa= 0.0393 g

7) Loading capacity of OVA when it only binds to the surface = 0.0393 g OVA/1 g silica= 3.93%
